# Radiofrequency Identification Marker–Guided Right S7 Segmentectomy for a Deeply Located Pulmonary Nodule: A Case Report

**DOI:** 10.70352/scrj.cr.26-0320

**Published:** 2026-07-10

**Authors:** Taichi Matsubara, Fumihiko Kinoshita, Naoya Iwamoto, Kazuki Takada, Tomoyoshi Takenaka, Tomoharu Yoshizumi

**Affiliations:** 1 Department of Surgery and Science, Graduate School of Medical Sciences, Kyushu University, Fukuoka, Fukuoka, Japan; 2 Department of Thoracic Surgery, Kyushu University Hospital, Fukuoka, Fukuoka, Japan

**Keywords:** preoperative marker, non-small cell lung cancer, lung segmentectomy, radiofrequency identification

## Abstract

**INTRODUCTION:**

The right S7 segment is the smallest pulmonary segment. Securing an adequate surgical margin during S7 segmentectomy can be challenging, particularly when the tumor is located more centrally. Radiofrequency identification (RFID) marking systems provide substantial benefits in detecting small nodules as well as ensuring an adequate resection margin from the marking site.

**CASE PRESENTATION:**

A 67-year-old man with a history of renal cell carcinoma was referred to Kyushu University Hospital with a 9-mm solid nodule located deep within the right S7 segment. Given its central and deep location, preoperative RFID marker placement was performed in a hybrid operating room under cone-beam CT guidance. Subsequently, thoracoscopic S7 segmentectomy was performed using a confronting upside-down video-assisted thoracoscopic surgery approach. The RFID probe enabled precise intraoperative localization of the tumor and continuous confirmation of its surgical margin from the intersegmental plane. Pathological examination revealed papillary adenocarcinoma and confirmed complete resection. The patient’s postoperative course was uneventful.

**CONCLUSIONS:**

RFID represents a valuable adjunct for segmentectomy of deeply or centrally located pulmonary nodules.

## Abbreviations

CBCT
: cone-beam CT
RFID
: radiofrequency identification
VAL-MAP
: virtual-assisted lung mapping
VATS
: video-assisted thoracoscopic surgery

## INTRODUCTION

Anatomical segmentectomy has emerged as an oncologically acceptable alternative to lobectomy in patients with early-stage non-small cell lung cancer.^1)^ However, ensuring an adequate surgical margin remains a critical concern, particularly for tumors located deep within the lung parenchyma. The right S7 segment is the smallest pulmonary segment, and securing an adequate surgical margin during S7 segmentectomy can be challenging. In such cases, precise intraoperative tumor localization is essential to achieve complete resection while preserving lung function.

Recent advances in localization techniques, including RFID marking systems, have provided new opportunities for accurate tumor detection and margin assessment. Herein, we report a case of right S7 segmentectomy for a deeply located pulmonary nodule wherein preoperative RFID marker placement enabled successful resection with an adequate surgical margin.

## CASE PRESENTATION

A 67-year-old man with a history of renal cell carcinoma was referred to Department of Thoracic Surgery, Kyushu University Hospital with an abnormal pulmonary nodule detected on CT. A 9-mm solid nodule was localized in the S7a subsegment (**Fig. 1A**). 3D CT revealed a pulmonary artery accompanying S7 branching from A8, with A4 arising slightly proximal to the bifurcation (**Fig. 1B**). The B7 bronchus branched anteriorly above the common basal vein and was divided into B7a and B7b (**Fig. 1C**). A distinct V7b branch could not be clearly identified on the preoperative CT images. 3D reconstruction demonstrated that the tumor was located only 11 mm from the S8 intersegmental plane (**Fig. 1D**), indicating that the surgical margin could become critically narrow if the intersegmental plane was created even slightly closer to the S7 side. In addition, because the lesion was located deep within S7 adjacent to the mediastinal side, direct palpation through the small incision used in the confronting upside-down VATS approach was expected to be difficult. Therefore, we decided to place an RFID marker preoperatively and perform S7 segmentectomy to facilitate precise intraoperative localization of the tumor and ensure an adequate surgical margin during intersegmental plane division.

**Fig. 1:**
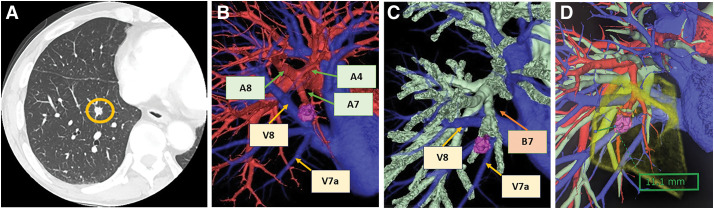
Preoperative radiological imaging and anatomical assessment of the right S7 segment. (**A**) CT demonstrating a 9-mm solid nodule (circle) located deep within the right S7a subsegment. (**B**) 3D CT showing the branching pattern of the pulmonary arteries, with A7 arising from A8 and A4 branching proximally. (**C**) B7 is close to V8, branching anteriorly above the common basal vein and dividing into B7a and B7b. (**D**) 3D CT reconstruction showing the tumor (the left end of the line) located 11 mm from the S8 intersegmental plane (the right end of the line).

RFID marking and subsequent segmentectomy were performed in a hybrid operating room. Under general anesthesia, with the patient in the supine position, a CBCT system was used to place the RFID marker as close as possible to the target lesion (**Fig. 2A**). After marker placement, CBCT was repeated during an inspiratory breath-hold with the positive end-expiratory pressure maintained, confirming that the deployed RFID marker was accurately positioned in close proximity to the tumor (**Fig. 2B**).

**Fig. 2:**
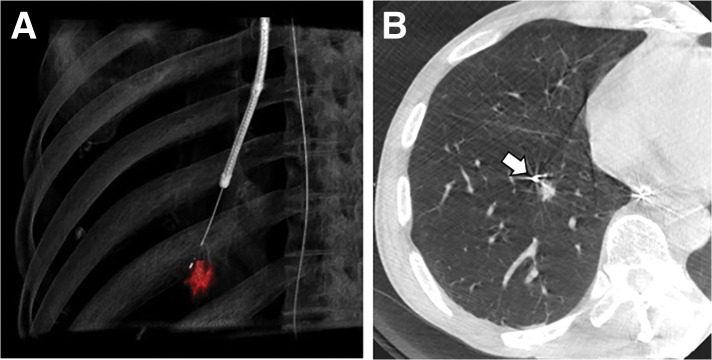
(**A**) CBCT-guided placement of the RFID marker in close proximity to the target lesion in a hybrid operating room. (**B**) Confirmation CBCT demonstrated accurate positioning of the RFID marker adjacent to the tumor. CBCT, cone-beam CT; RFID, radiofrequency identification

S7 segmentectomy was performed using the confronting upside-down VATS approach. The tumor location was identified using an RFID probe, and the corresponding point on the lung surface was marked. The interlobar fissure was dissected to expose the common basal pulmonary artery, and the branching patterns of A4, A7, and A8 were confirmed. After completing a fissure between the middle and lower lobes, the A7 was divided using a stapler. The posteriorly located B7 was then identified and divided using a stapler. The B7 stump was retracted dorsally to secure a sufficient distance from the common basal vein and V8, facilitating division of the intersegmental plane between S7 and S8–10. During dissection around V8, a thin V7b branch arising from V8 was identified and cut using a sealing device (Ligasure; Medtronic, Mansfield, MA, USA).

After marking the intersegmental line of S7 using indocyanine green fluorescence imaging, the intersegmental plane was divided using a stapler. The stapler was placed on the hilar side of the upper portion of the intersegmental vein, the surrounding lung parenchyma was grasped, and the stapler position was adjusted before transection. During this process, the RFID probe was used to confirm that the tumor was included within the resected specimen and that an adequate surgical margin from the resection stump had been secured (**Fig. 3A**). Thereafter, S7 segmentectomy with mediastinal lymph node dissection was performed (**Fig. 3B**). The specimen was not submitted for intraoperative frozen-section pathological examination. Operation time, including RFID placement, was 188 min, and the total blood loss was 5 mL. The postoperative course was uneventful. Pathological examination confirmed primary lung adenocarcinoma with a predominantly papillary pattern, with a maximum size of 9 mm and a surgical margin distance of 10 mm.

**Fig. 3:**
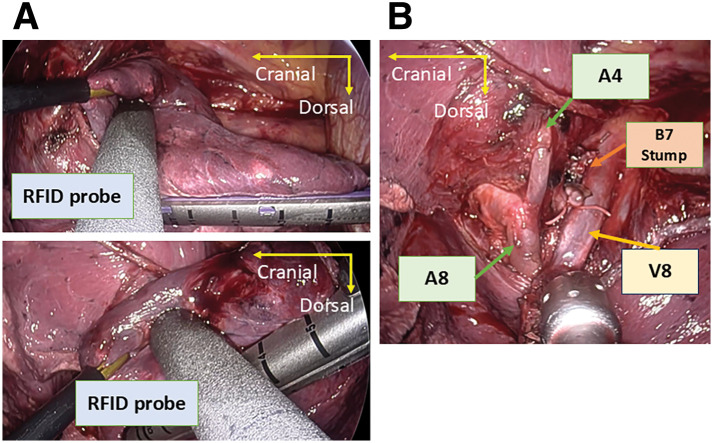
(**A**) Repeating intraoperative use of the RFID probe to localize the tumor and confirm its inclusion within the resected specimen, ensuring an adequate surgical margin. (**B**) Operation view after S7 segmentectomy, with V8 preserved. RFID, radiofrequency identification

## DISCUSSION

We utilized an RFID-based marking technique to perform S7 segmentectomy for a tumor located deep within the S7 segment, achieving an adequate resection margin while contributing to maximal preservation of the lung parenchyma. Segmentectomy is the optimal surgical option for small peripheral lung tumors.^2)^ In this “segmentectomy era,” the present case highlights 2 critical aspects in performing S7 segmentectomy: the inherent surgical limitation of this smallest and deeply located segment, and the potential of RFID-based localization to overcome this difficulty.

First, the anatomical complexity of the right S7 segment poses a significant obstacle in achieving an adequate surgical margin. The S7 segment is widely recognized as the smallest pulmonary segment and is located deep within the basal segmental structure, in close proximity to the common basal vein and adjacent segmental bronchi. This anatomical constraint restricts the surgeon’s ability to extend the resection line without compromising adjacent segments. Deeply located tumors, particularly in basal segments, are associated with increased technical difficulty during segmentectomy because of the depth of the target lesion and the complexity of the segmental anatomy.^3)^ In such situations, the inability to accurately localize the tumor intraoperatively may result in insufficient surgical margins.

The importance of securing an adequate surgical margin during segmentectomy is well established. Several studies have demonstrated that local recurrence is strongly associated with insufficient margins. Schuchert et al. emphasized that both the absolute margin distance and the margin-to-tumor ratio are critical determinants of local control following segmentectomy.^4)^ Furthermore, a margin distance of at least 2 cm or greater than the tumor diameter is considered desirable to minimize the risk of recurrence.^5,6)^ These findings underscore the importance of precise tumor localization and margin assessment, particularly for deeply located lesions.

Second, our case demonstrated that RFID-based marking can play a pivotal role in overcoming anatomical and oncological challenges. In recent years, several localization techniques, including hookwire placement, dye marking, and VAL-MAP, have been developed to facilitate resection of small or nonpalpable pulmonary nodules.^7–9)^ However, these techniques have inherent limitations. For example, although VAL-MAP provides valuable surface marking, its ability to accurately reflect tumor depth is limited, particularly in deeply located nodules. This limitation is critical in segmentectomy, where 3D spatial understanding is essential to ensure an adequate margin.

In contrast, RFID offers distinct advantages. The ability to detect the marker intraoperatively using a probe enables real-time 3D tumor localization.^10)^ In the present case, the RFID system allowed accurate identification of the tumor location on the lung surface and continuous confirmation of its spatial relationship with the resection line during parenchymal division. By repeatedly confirming the tumor position relative to the stapling line, we ensured that an adequate margin was maintained throughout the procedure.

Furthermore, the significance of the present case extends beyond overcoming the limitations of conventional localization techniques. The indications for segmentectomy are expected to expand from peripheral lesions to more centrally located pulmonary nodules. However, achieving an adequate surgical margin in centrally located tumors remains technically challenging because the resection line is constrained by complex segmental anatomy and proximity to hilar structures. In the present case, the tumor was located only 11 mm from the S8 intersegmental plane and was deeply situated on the mediastinal side of S7, making direct palpation difficult during the confronting upside-down VATS approach. RFID guidance enabled precise intraoperative localization of the lesion and provided real-time confirmation of its relationship to the intersegmental plane, thereby facilitating confident parenchymal division while maintaining an adequate surgical margin. Therefore, the value of RFID technology in this setting lies not only in tumor localization but also in supporting margin-oriented anatomical segmentectomy for deeply located central lesions. This approach may contribute to the future expansion of segmentectomy indications to centrally located pulmonary nodules, where margin assessment remains 1 of the major technical challenges.

## CONCLUSIONS

S7 segmentectomy for deeply located tumors remains technically demanding owing to anatomical constraints and difficulty in securing adequate surgical margins. Our case demonstrates that RFID-based marking offers a promising solution by enabling precise real-time tumor identification and margin assessment. This technique may facilitate safe and effective complex segmentectomies in challenging cases, thereby expanding the role of segmentectomy.

## DECLARATIONS

### Availability of data and materials

Data will be made available upon reasonable request to the corresponding author.
